# Return to work in head and neck cancer survivors: an exploratory multimethod study at a cancer centre in Santiago, Chile

**DOI:** 10.3332/ecancer.2025.1847

**Published:** 2025-02-13

**Authors:** Ximena Mimica, Loreto Fernández González, Jorge Sapunar, Felipe Contreras, Matías Lavín, O Gustavo Vial, C Gustavo Vial, Daniel Ledezma, Luis Marín, David Cohn

**Affiliations:** 1 Head and Neck Service, Department of Surgery, Instituto Oncológico Fundación Arturo López Pérez, Santiago 7500691, Chile; 2 Cancer Research Department, Instituto Oncológico Fundación Arturo López Pérez, Santiago 7500691, Chile; 3 Dalla Lana School of Public Health, University of Toronto, 155 College St, Toronto, ON, Canada; a https://orcid.org/0000-0002-7105-5143; b https://orcid.org/0000-0001-5026-6438; † Contributed equally to this work as co-authors.

**Keywords:** cancer survivors, head and neck neoplasms, return to work, employment

## Abstract

**Objective:**

Head and neck cancer (HNC) survivors may suffer from functional and psychosocial impairment, and thus, return to work (RTW) often poses challenges. A paucity of evidence on this subject exists in Chile and the region. The aim of this paper is to describe and characterize the RTW of HNC survivors treated at a cancer centre in Santiago.

**Methods:**

This study employed an exploratory, cross-sectional design, with a multimethod, quantitative approach. Surgically treated patients with HNC between 2016 and 2022 were invited to participate. Clinical and sociodemographic data were statistically analysed to establish associations with RTW. Participants were surveyed about their process of RTW and income variation.

**Results:**

Of the 120 patients identified, 53 agreed to participate. Twenty-nine patients (55%) were men with a median age of 56 years. The most frequent location of the cancer was the oral cavity (62%), and 85% of them had locally advanced tumours. Thirty-seven patients (70%) were working at diagnosis, of which 25 (68%) were men. Twenty-nine (78%) survivors returned to work after treatment. Being a woman was significantly associated with a lower chance of RTW (*p* = 0.046). No association was found between disease status, tumour location or treatment received and RTW. Of those who resumed working, a third had less income. Job accommodations were made on a case-by-case basis. A third of the survivors decreased their workload.

**Conclusion:**

Being a woman was associated with less RTW. Future interventions should provide support in reintegration into the workplace. This study constitutes the first published data on RTW in Chilean patients.

## Background

In 2019, the global cancer incidence, excluding nonmelanoma skin cancers, was estimated to be 17.2 million, generating an estimated 250 million disability-adjusted years of life [1]. In Chile in 2020, the estimated incidence of cancer was 54.227 cases per 100,000 people per year, and cancer mortality was 28.584 per 100,000 people per year [2], becoming the highest cause of death in the country. Particularly for cancers located in the head and neck region, excluding the thyroid, it was estimated that 818 new cases were diagnosed and 418 people died from this cause.

Head and neck cancer (HNC) includes a group of malignancies located at different sites in the upper aerodigestive tract, including those located in the oral cavity, oropharynx, larynx and paranasal sinuses. Management consists of a single-modal or multimodal treatment that involves surgery, radiotherapy and systemic therapy. Psychosocial impact is common due to functional impairment and changes in appearance [3]. Despite the growing effectiveness of available treatments, the management of physical, psychosocial and economic sequelae often presents important challenges that involve health providers during treatment and the social context during survivorship as well. Though HNC historically affected predominantly older people who were smokers or suffered from alcohol abuse, in the last two decades an epidemiological shift has been observed with a higher incidence of papillomavirus-related cancers. These patients tend to be younger (around 40 years old), without comorbidities and actively working [4].

In this scenario, occupational reintegration has been considered a key component of survivorship [5]. Return to work (RTW) provides not only economic income but is also a part of a person’s identity, allows social interaction and provides purpose as well as a sense of normality. RTW is a key challenge during survivorship for cancer survivors, regardless of the tumour location. Cancer survivors reportedly face unemployment up to 1.4 times more than people without a cancer diagnosis [6]. Unemployment, early retirement and informality worsen quality of life and the severity of the financial toxicity associated with the disease [7]. Evidence shows that RTW in HNC survivors can range from 32% to 90%, who often lack supportive work environments [8]. In addition, HNC survivors can suffer from social stigma due to their diagnosis and the functional and physical impairment from treatments, which often affects their participation in social life, including RTW, significantly more than other cancer survivors [3, 9].

A recent analysis from GLOBOCAN indicated that the incidence of cancer has been steadily rising in South America, and it is predicted to continue to increase due to higher life expectancy and adoption of Western lifestyles [10]. Specifically in Chile, cancer control measures have been put in place in recent decades, including a National Cancer Plan launched in 2020 [11]. However, survivorship remains an understudied area and published data on RTW is nonexistent to date. Moreover, no specific health programs prevent, manage and/or palliate HNC patients, as opposed to cancers with higher incidences that have access to the Explicit Health Guarantees program in the country. In this context, it is reasonable to expect that Chilean HNC survivors experience substantial difficulties when RTW is exposed to significant financial distress and job precariousness.

In summary, there is insufficient evidence regarding cancer survivors and RTW in Chile and South America. Moreover, there is no published data on Chilean HNC survivors, and the clinical and sociodemographic factors associated with RTW and the impact of the disease on their working status and income. Thus, this article intends to describe and characterize the RTW of HNC survivors treated at a cancer centre in Santiago, Chile.

## Methods

### Patients

We retrieved the medical records of patients older than 18 years with head and neck tumours who were treated surgically with microsurgical flap reconstruction between 2016 and 2022 at the Instituto Oncológico Fundación Arturo López Pérez, a nonprofit private cancer centre located in Santiago, Chile. Demographic information (age, sex and insurance), tumour details (anatomical site and stage of disease) and treatments received (type of microsurgical flaps, radiation therapy and adjuvant chemotherapy) were obtained. Patients treated at other institutions were excluded. This study is nested in an exploratory project on the stigma experiences of HNC survivors. This project was approved by the research ethics board of the institution according to current regulations (study No. 2019-A-001).

### Study design

The present study corresponds to a nonexperimental, cross-sectional design, with a multimethod, quantitative approach. Clinical and sociodemographic data were statistically analysed to establish associations between variables and RTW. In addition, the study participants were surveyed to inquire about their working status and characterize their process of RTW and income variation. This multimethod approach was chosen to provide the most comprehensive description of the problem [12]. Though the cancer centre provides long-term follow-up to survivors, RTW is not measured or documented in a standardized way. Therefore, the survey component allowed the research team to explore participants’ accounts of RTW, capturing the variety of trajectories experienced by them, while also simultaneously enriching the statistical analysis of the numerical data.

Eligible patients were contacted by telephone by a member of their treatment team (XM), and a structured interview was used. After consenting to participate, they were asked the following questions.

1. Were you working at the time of the diagnosis? Yes/no
2. If yes, describe your job category and employment status.
  - Did you return to your job? Yes/no
  - Did you have to modify your job description and/or workload? (Yes/no). Describe.
  - Was your income impacted by the cancer diagnosis? (Yes/no). Describe.

### Data analysis

Participants’ answers were transcribed and examined through content analysis. Two members of the research team (XM and LF-G) read the transcripts multiple times to become familiar with the data. Questions 1 and 2a and the first part of 2b and 2c that prompted a dichotomous response were grouped and quantitatively analysed. The open-ended sections of 2b and 2c were grouped into predefined categories created by both researchers to describe the frequency and main features of how participants RTW. Answers that were considered as exemplifying the main changes in working status and/or employment were selected to illustrate further some of the individual pathways.

Clinical and demographic data were analysed to establish associations between these variables and RTW. For univariate analysis, a *χ*^2^ test was used for categorical variables and *t*-test was used for continuous variables. The Cochrane–Armitage test was used to evaluate trends in ordinal variables in multiple categories. The significance level was 0.05. A multivariate analysis was also performed using logistic regression.

## Results

### Clinical description of the sample

We identified 120 patients with HNC treated with microsurgical flap reconstruction between 2016 and 2022. Of these, 53 patients agreed to participate. Twenty-nine participants (55%) were men, and the median age was 56 years (range 19–81 years). Thirty-seven participants (70%) had public insurance. The most frequent location of the primary tumour was the oral cavity in 33 (62%) patients, in which the most affected anatomical subsite was the tongue (14 patients, 26%), followed by paranasal sinuses affected in 7 (13%) patients. The most common microsurgical flap reconstruction was the anterolateral thigh in 19 patients.

Forty-five patients (85%) presented with locally advanced T3 and T4 tumours, and 41 (77%) patients received multimodal treatment (Table 1).

### Employment status at diagnosis and RTW

Of the 53 patients, 37 (70%) were working at diagnosis, of whom 25 (68%) were men. Twenty-nine of the actively working survivors at diagnosis returned to work after treatment (78%), of whom 22 (69%) were men and 7 (31%) were women. Seventeen had public insurance (59%) and 12 (41%) had private insurance.

The average age of the eight patients who did not RTW was 55.6 years (a range of 47–63 years). Six of them had tumours in the oral cavity, two of them had tumours in the paranasal sinuses and five patients presented with locally advanced disease at the time of diagnosis requiring extensive surgeries, such as bilateral maxillectomy, total glossectomy or orbital exenteration.

A comparison between patients according to their work status post-treatment showed no differences in age, T stage, type of reconstruction or the need for multimodal treatment (Table 2). Female sex was associated with less RTW in the univariate and multivariate analysis (OR = 0.12; CI 0.014–0.961,* p* = 0.046).

### Characterization of RTW

**Figure 1.**
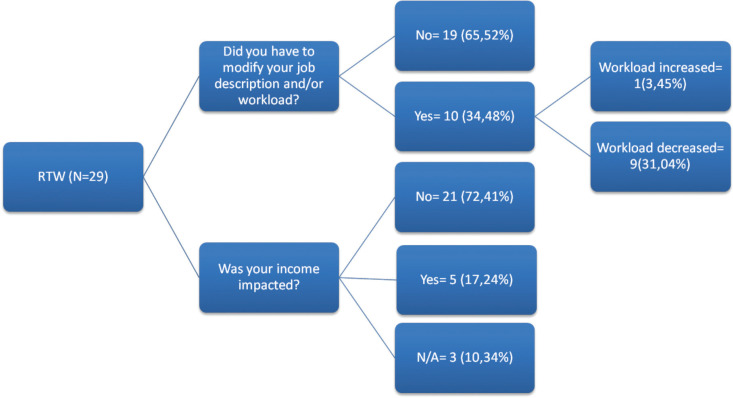
Changes in RTW in HNC survivors.

Of the group of 29 survivors who returned to work, 10 (35%) changed their job or returned to a different position within their workplace. Of these, only one participant reported that his working hours had increased due to a shift to self-employment. The other nine survivors (31%) reported that their workload decreased or their functions were changed to allow them to return to the same workplaces they had before the diagnosis. In terms of income, 21 participants (72%) indicated no change in their income due to cancer, five participants (17%) reported a decrease in income and three participants (10%) preferred not to answer (Figure 1). Examples of changes in employment reported by the survivors on their RTW can be found in Table 3.

## Discussion

RTW is a fundamental aspect of reprising previous social roles for cancer survivors. Although it has been documented that this group is less likely to be employed compared to the healthy population [7] in patients treated for HNC, the proportion of patients returning to work is even lower than those experiencing other cancers [9]. Moreover, RTW varies widely (32%–90%) between the different countries and is influenced by idiosyncratic aspects and social policies [13, 14].

Currently, in Chile, treatment for HNC is not part of the Explicit Health Guarantees defined by the Ministry of Health, which produces great variability in the timely access to treatments, the availability of these throughout the country and the standardized presence of quality supportive care, including rehabilitation. This affects patients’ ability to access cancer treatments at earlier stages of the disease, poses a significant risk of financial toxicity and affects the chances of returning to their previous jobs and in similar working conditions. Moreover, current local legislation punishes the dismissal of patients with cancer diagnoses but does not force the employer to generate work modifications for the survivor’s return. As shown in our study, over a third of the survivors that RTW did so in different working conditions, and the participants’ responses depicted that these modifications tended to happen on a case-by-case basis.

All but one of the participants that RTW reported that their working hours decreased or their tasks were modified to meet their new requirements, in accordance with published experiences in which the percentage of patients who reduced their working hours is between 44% and 32% [15, 16].

The data show that almost a quarter of the patients actively working at the time of diagnosis did not RTW. In addition, a third of the participants’ RTW reported an impact on income compared to status prior to diagnosis. These data shed light on possible psychosocial and financial needs that undermine survivors’ quality of life. Financial needs have an important impact on RTW, and it has been reported that when access to sick leave is severely restricted, patients tend to RTW earlier despite not feeling ready to do so [17, 18]. In the case of patients without paid leave, the recovery time causes younger patients to leave their jobs or retire if they are close to retirement age [19].

Our study shows that being female is associated with a lower chance of returning to work after a diagnosis of HNC. This contrasts with what has been published in international literature, in which most studies did not find a significant association between sex and RTW. Two publications showed disparate associations: the results by Agarwal *et al* [15] reported that women were more likely to RTW compared to men, whereas Check *et al* [19] showed that women were 35% more likely to stop working compared to men. Future studies on the subject should clarify whether this is related to the different retirement ages defined in Chile by sex (60 years for women and 65 for men) and thus structurally determined, and whether this is a voluntary decision or a decision pressured by insurers and pension fund managers.

Our sample did not find a statistically significant association between a more advanced disease and less RTW as in other studies [20, 21], possibly because the patients had in common receiving surgery involving a free flap, and most of them (85%) were diagnosed at an advanced stage of their disease. The anatomical site also had no significant effect, even though up to 62% of patients had tumours in the oral cavity, particularly the tongue, affecting speech.

This present paper has strengths and limitations. To our knowledge, this is the first published data on the subject and in this group of cancer survivors in the country and the region, which constitutes a valuable contribution to the emerging understanding of the epidemiology of cancer survivors, specifically those diagnosed with HNC. Furthermore, this cancer centre is the largest of its kind in Chile and treats patients with public and private insurance, including patients who are financially and socially vulnerable. All surveys were conducted by a surgeon with extensive experience as a clinician with these patients. Methodologically speaking, we designed a multimethod study collecting quantitative and qualitative data to describe the research problem comprehensively. Content analysis was chosen as the most appropriate tool to add analytical depth to the clinical and epidemiological variables.

## Study limitations

Our study has several limitations. The sample size might not be adequate to draw strong statistical conclusions. However, it is important to highlight that HNC diagnoses are less prevalent than other types of cancer and their diagnosis at later stages affects survivorship, which might affect RTW rates compared to other cancers. In addition, our study was conducted by phone, which might be challenging for this group of survivors. In addition, patient contact databases may not have been updated in the clinical records, affecting the response rate. Multiple factors have not been analysed in this study that could potentially influence RTW, such as the concern for physical appearance, the presence and quality of a support network, the length of sick leave, detailed information on the job positions and the type of contract that the participants had before and after the cancer diagnosis.

## Clinical implications

HNC survivors exhibit a distinct profile of side effects, and surgical procedures for HNC target an anatomically complex area, sometimes with long-term consequences such as oral dysfunction, speech and swallowing problems. This region is also highly exposed, as our face serves as our primary means of presenting ourselves and interacting with others. According to a published study, patients felt that they were more conscious of people looking at them and judging their speech, which affected their self-confidence [22]. While oncological surgeries in other tumour locations can also have high morbidity, those areas are typically easier to conceal, such as gastrointestinal or genitourinary cancers. The side effects we describe do not only refer to the biological dimensions of the disease and treatments but also include social phenomena such as stigma and shame. Moreover, as quoted by the HNC Survivorship Consensus Statement, HNC survivors have a high psychosocial burden being suicide a critically important issue. HNC survivors are twofold more likely to die from suicide than patients with other types of cancer [23]. Therefore, this warrants a deeper investigation into their post-treatment challenges and their interaction with their social surroundings. Recognize that RTW linked to an individual’s sense of identity can facilitate the development of effective national policy aimed at reintegrating them into the workforce.

## Conclusion

Almost a quarter of the patients actively working at the time of an HNC diagnosis did not RTW. Of those who did resume working, a third reported an impact on income compared to status prior to diagnosis. Our study shows that being a woman was associated with a lower chance of returning to work compared to men. No association was found with disease status, tumour location or treatment received. Job accommodations were made on a case-by-case basis. Survivors that RTW often decreased their workload or had to change the tasks they performed to be able to work again. Future interventions should aim to identify at-risk patients to provide support for reintegration into the workplace. To our knowledge, this study constitutes the first published data on the subject in Chilean patients and in the region.

## Conflicts of interest

None of the authors have any conflicts of interest or financial disclosures.

## Funding

This research received no external funding.

## Figures and Tables

**Table 1.** Patients’ characteristics.

Age average (range)	56 (19–81)
Sex (%) • Males • Females	29 (55) 24 (45)
Insurance (%) • Public • Private	37 (70) 16 (30)
Anatomical site (%) • Oral cavity • Paranasal sinuses • Hypopharynx • Oropharynx • Salivary glands • Skin	33 (62) 7 (13) 4 (8) 2 (4) 2 (4) 5 (9)
*T* Stage (%) • T0 • T1 • T2 • T3 • T4	5 (9) 6 (11) 7 (13) 12 (23) 23 (43)
*N* Stage (%) • N0 • N+	42 (79) 11 (20)
Free flap (%) • Anterolateral thigh • Radial forearm • Fibula flap • Jejunal flap • Latissimus dorsi	19 (35) 16 (30) 13 (25) 4 (8) 1 (2)
Tracheostomy (%) • No • Yes	10 (19) 43 (81)
Treatment (%) • Surgery • Surgery + RT† • Surgery + CRT‡ • Surgery + Chemotherapy	12 (23) 28 (53) 12 (23) 1 (2)

†RT radiotherapy, ‡CRT chemoradiotherapy

**Table 2.** Comparison between patients according to their work status.

Factor	RTW N = 29	No returning to work N = 8	p-value
Age average	52	56	0.479
Sex (%) • Male • Female	22 (76) 7 (24)	3 (38) 5 (62)	0.040
Insurance (%) • Public • Private	17(81) 12(75)	4 (19) 4 (25)	0.660
Anatomical site (%) • Oral cavity • Paranasal sinus • Hypopharynx • Oropharynx • Salivary gland • Skin	18 (62) 3 (10) 2 (7) 1 (3) 1(3) 4 (14)	6 (75) 0 (0) 0 (0) 1 (12) 0 (0) 1 (12)	*0.735 **0.606
T stage (%) • T0 • T1 • T2 • T3 • T4	4 (16) 4 (16) 2 (8) 5 (20) 10 (40)	0 (0) 0 (0) 0 (0) 2 (29) 5 (71)	*0.388 **0.933
N Stage (%) • N0 • N+	19 (73) 7 (27)	7 (100) 0 (0)	0.495
Free flap (%) • Anterolateral thigh • Radial forearm flap • Fibula free • Jejunal • Dorsal	9 (31) 7 (24) 9 (31) 3(10) 1(4)	4 (50) 2 (25) 2 (25) 0 (0.00) 0 (0.00)	0.768
Tracheostomy (%) • No • Yes	3 (12) 23 (88)	2 (29) 5 (71)	0.265
Multimodal treatment (%) • No • Yes	7 (27) 19 (73)	0 (0) 7 (100)	0.122

*Ji2 **Cochran-Armitage

**Table 3.** Examples of changes in employment status and/or job description.

Changes in employment/job	Example
Reduction in working hours/workload	Change of contract from full time to part time
Increase in working hours/workload	Worker shifted to self-employment, with a higher workload
Modification of job position (tasks)	School teacher is no longer teaching in-class lectures and is now in charge of the school library
Change in employment status	Worker negotiates his firing with the employer to obtain financial compensation benefits
